# Experimental study of pore-scale flow mechanism of immiscible CO_2_ flooding under in-situ temperature-pressure coupling conditions

**DOI:** 10.1371/journal.pone.0321527

**Published:** 2025-04-01

**Authors:** Tingting Li, Suling Wang, Jinbo Li, Kangxing Dong, Zhennan Wen

**Affiliations:** 1 School of Mechanical Science and Engineering, Northeast Petroleum University, Daqing, China; 2 Equipment Department, Kingchem (Liaoning) Life Science Co., Ltd., Fuxin, Liaoning, China; University of Sharjah, UNITED ARAB EMIRATES

## Abstract

The flow mechanism of CO_2_ flooding serves as the theoretical foundation for examining the synergic integration of oil recovery and CO_2_ storage. Immiscible CO_2_ flooding has attracted considerable research attention due to its simplicity and cost-efficiency. However, minimal studies are available regarding the flow characteristics and EOR mechanism of immiscible CO_2_ flooding in in-situ temperature-pressure coupling conditions at the pore scale. Therefore, this study employed a modified high-temperature, high-pressure microfluidic system to simulate the in-situ CO_2_ and water injection processes in a combined temperature-pressure environment and analyze the multiphase flow characteristics in the pores. The injection rate, displacement pressure difference, displacement efficiency, and residual oil distribution were quantitatively analyzed at different pressures. The results indicated higher residual oil clustering after water flooding at the same injection rate. CO_2_ flooding significantly reduced residual oil clustering and enhanced the oil flooding effect. The multiphase flow dynamics, type of remaining oil in different injection conditions, and flow characteristics of immiscible CO_2_ flooding were determined. A higher confining pressure interrupted the CO_2_ flow, which destabilized the displacement front increased the recovery efficiency by 12.9%. Furthermore, a higher injection rate and displacement pressure increased the recovery efficiency by 24.9% and 6.1%, respectively.

## Introduction

The excessive utilization of energy sources such as oil, coal, and natural gas has increased the global CO_2_ levels, which has intensified the greenhouse effect [1,2] and led to glacier melting, lower agricultural output, and frequent climate disasters [3]. Therefore, reducing the atmospheric CO_2_ levels is crucial for alleviating the greenhouse effect. Of the available carbon capture, utilization, and storage (CCUS) technologies, CO_2_ flooding is considered safe and effective for enhancing oil recovery and reducing CO_2_ emissions [4,5]. Injecting liquid or supercritical CO_2_ into reservoir oil fields for crude oil displacement enhances the oil recovery rate and enables CO_2_ sequestration, offering dual advantages [6–14]. Most CO_2_ flooding methods used globally for enhancing oil recovery adopt miscible flooding technology. However, this method is less effective in China due to the reservoir depths and heavy components of crude oil. Therefore, near-miscible and non-miscible flooding have gradually been applied in domestic oilfields to increase oil production [15]. Non-miscible flooding has become an attractive alternative for oil recovery due to advantages such as lower required injection pressure, cost-efficiency, and higher operational feasibility [16,17].

Hamza, A. et al. examined the influence of various factors on the efficacy of CO_2_ displacement and recovery in sandstone and carbonate reservoirs at the nanoscale level, focusing on evaluating pilot CO_2_ sequestration in the field [18]. They also conducted geological feature studies in an oilfield demonstration area to analyze residual oil distribution and gas migration after CO_2_ flooding, establishing an optimized evaluation method for CO_2_ flooding sequestration [19]. Li et al. conducted core-flooding experiments to quantitatively characterize the efficacy of CO_2_ injection in enhancing oil recovery and sequestration [20]. Since CO_2_ and crude oil molecule diffusion, the nanopore effect, and adsorption affect CO_2_ storage and transport during CO_2_ flooding, it is crucial to consider the influence of the multiphase medium at the pore scale [21]. Therefore, understanding the microscale flow mechanisms of CO_2_ flooding is essential for developing reservoir scale and field applications. Andrew, M. et al. scanned reservoir environments via X-ray microtomography to quantify the captured residual CO_2_ after activation and reuse [22]. Abdulla Alhosani et al. introduced in-situ CO_2_ injection into three-phase miscible flooding to examine the oil recovery mechanism, pore occupancy rate, and interfacial areas [23]. However, when CO_2_ is continuously injected as a fluid into the reservoir pores, capillary resistance causes it to flow through larger holes, preventing crude oil displacement in smaller and microscopic pores, consequently affecting the oil recovery rate [24–26]. Macroscopic core-flooding experiments and reservoir-scale studies cannot directly visualize the fluid flow in the porous medium. Therefore, experimental microscopic visualization techniques are employed to analyze the fluid flow at the pore scale and the CO_2_ flooding flow mechanisms [27–30]. This technique describes the near-real dynamic process of oil displacement at the pore scale to analyze its fluid flow mechanism [31]. However, conventional microfluidic devices are generally unable to withstand the harsh conditions in high-temperature and high-pressure reservoirs [32]. Therefore, the temperature and pressure of the experimental system are optimized to ensure appropriate in-situ thermal and pressure conditions [33]. Despite extensive research on fluid flow during CO_2_ flooding, minimal studies are available regarding the mechanism behind in-situ CO_2_ flow in microscopic pores in reservoir environments. Therefore, it is essential to investigate the mechanism behind the pore scale flow during immiscible CO_2_ displacement in an in-situ reservoir environment in combined temperature and pressure conditions. Image J gray scale analysis was performed to assess the CO_2_ injection rate, displacement pressure difference, and CO_2_ flooding oil recovery at different confining pressures to quantitatively analyze the influence of these factors on residual oil distribution. This method prevents the errors caused by microflow measurements and facilitates the real-time calculation of multiphase fluid dynamics. The results establish a theoretical foundation for developing enhanced immiscible CO_2_ flooding oil recovery technology.

## Experimental

### 2.1. Material preparation

**Fig 1:**
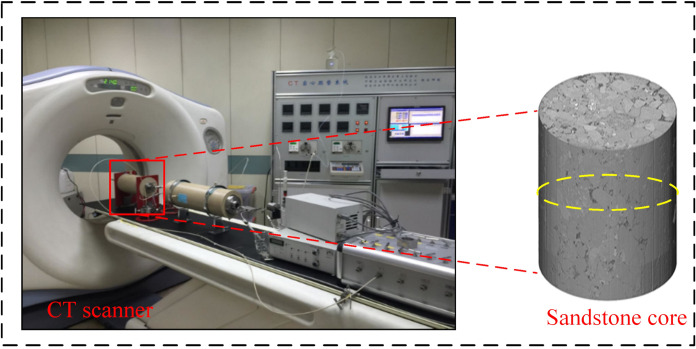
The CT scan of the core.

**Fig 2:**
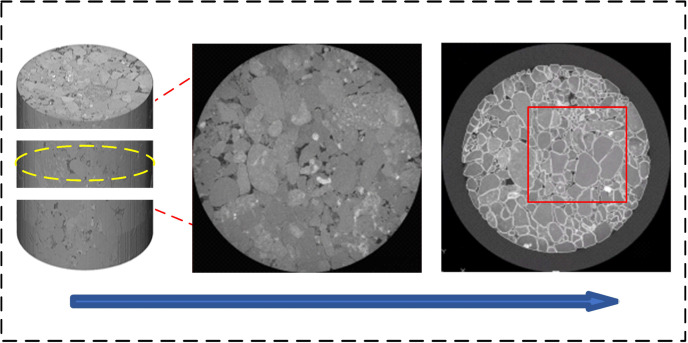
The extraction of the pore structure via the binarization of the target slices.

**Fig 3:**
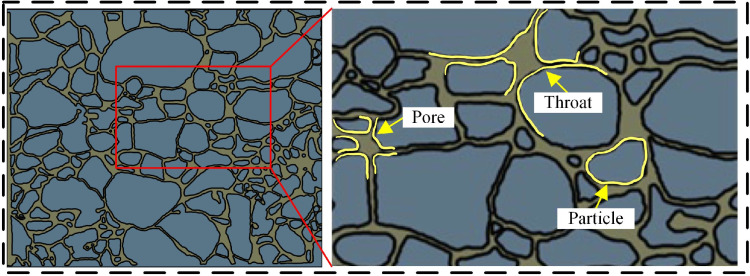
The representative areas of the section.

**Fig 4:**
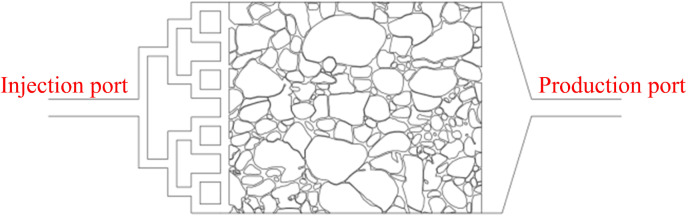
The glass-etched chip.

Sandstone samples were collected from a coring well in a reservoir in the Yanchang Oilfield. The reservoir temperature in the selected section was 80°C and the formation pressure was 15 MPa. Micrometer CT scanning was used to obtain 3D images of the sandstone (Fig 1), which were imported into Avizo for binarization to extract the pore structure of the core and select a suitable pore structure cut surface (Fig 2). The representative area of the cut surface (Fig 3) was etched out the pore structure on optical glass. Table 1 shows the basic parameters of the glass etching chip (Fig 4).

**Table 1:** The basic physical properties of the core samples.

Physical parameter	Porosity	Permeability (mD)	Throat size (um)	Model size (mm)
**Glass-etched chips**	9.6%	0.94	15 ~ 170	30 mm × 30 mm

The experimental simulation oil was prepared using the natural gas composition sourced from the field, the original gas-oil ratio, a CO_2_ purity of 99.99%, and a crude oil and CO_2_ MMP of 17.8 MPa. The properties are shown in Table 2.

**Table 2:** Simulated oil properties.

Simulated oil properties	Gas-oil ratio (m3/m3)	Viscosity (mPa·s)	Density (g/cm3)
**Parameter**	60	4.87	0.839

### 2.2. Experimental apparatus

**Fig 5:**
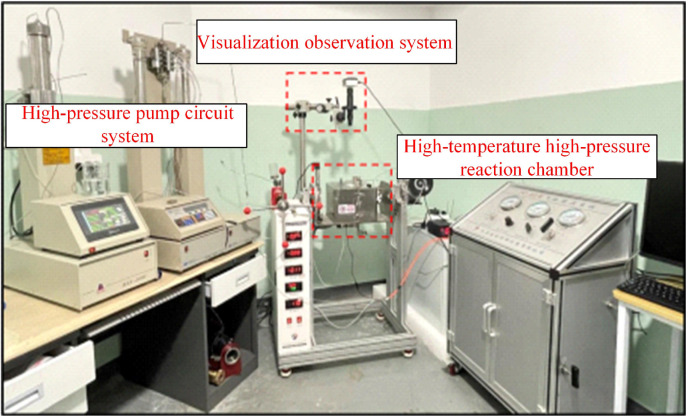
The experimental high-temperature, high-pressure microfluidic visualization system.

**Fig 6:**
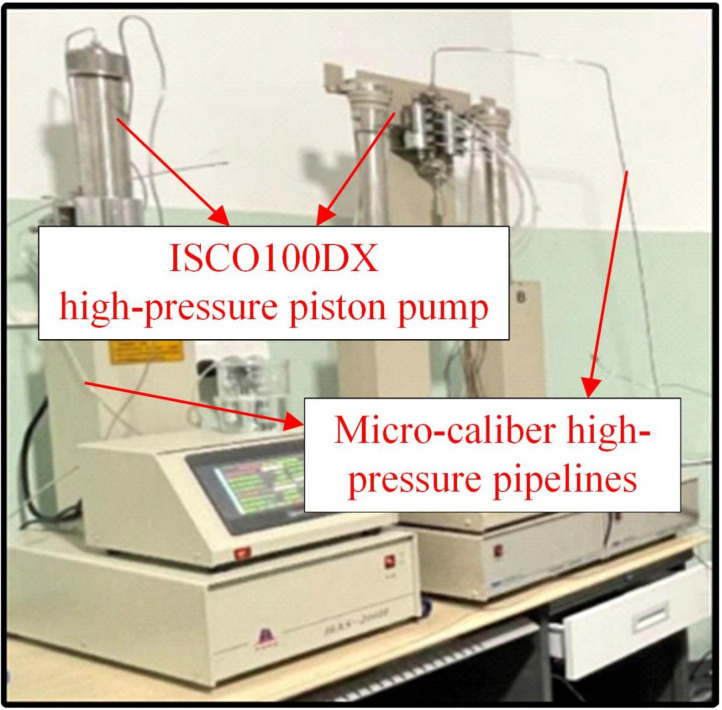
A diagram of the high-pressure pump system.

**Fig 7:**
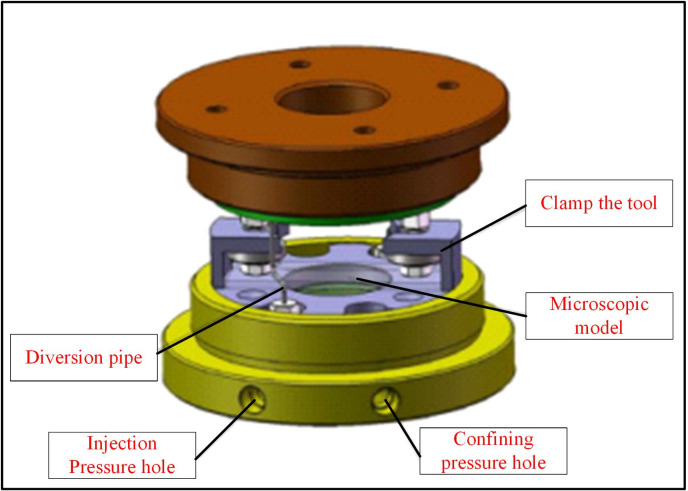
A schematic diagram of the high-pressure reactor device.

**Fig 8:**
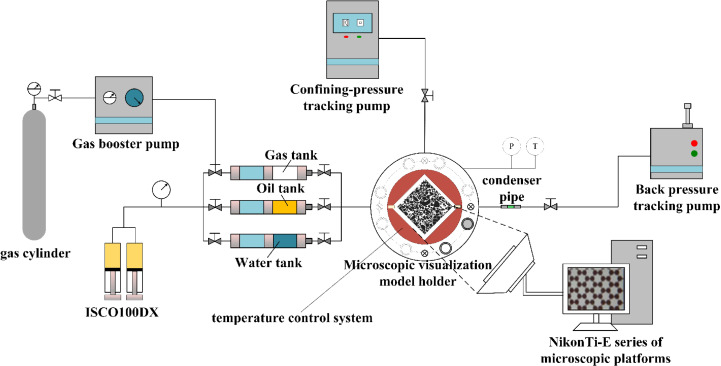
The experimental high-temperature, high-pressure microfluidic visualization system.

Fig 5 shows the high-temperature, high-pressure microscopic visualization experimental system. It mainly comprised three parts: the high-pressure pump circuit system, the visualization observation system, and the high-temperature, high-pressure reaction chamber. The high-pressure pump circuit system consisted of micro-caliber high-pressure pipelines and an ISCO100DX high-pressure piston pump (Fig 6). The piston pump had a capacity of 100 mL, showing a maximum pressure of 68.95 MPa and a pressure accuracy of 0.5% FS. It was capable of precisely maintaining a constant micro-flow rate, pressure, and programming. Simple settings were used to facilitate linear or stepwise system pressure changes. The confining-pressure tracking pump was used to adjust the confining pressure of the glass-etched chip to prevent excessive pressure differences between the interior and exterior, possibly damaging the chip. This pump was also employed to facilitate miscible and immiscible CO_2_ displacement by regulating the exit pressure of the chip. The Nikon Ti-E series microscopic platform and the microscopic model were used for system visualization. The microscopic platform presented a maximum resolution of 0.83 µm, enabling clear observation of the oil and CO_2_ flow processes in the glass-etched chip. The high-temperature, high-pressure reaction chamber consisted of a high-pressure reactor (Fig 7) and a temperature control system. To ensure the normal operation of the microscopic visualization window, a maximum working pressure and temperature of 25 MPa and 120°C were used for the experimental high-temperature, high-pressure microscopic visualization system. This satisfied the operational safety and stability requirements of the maximum pressure and temperature conditions of 13 MPa and 80°C in this paper. Fig 8 shows a schematic diagram of the system.

### 2.3. Experimental procedure

**Fig 9:**
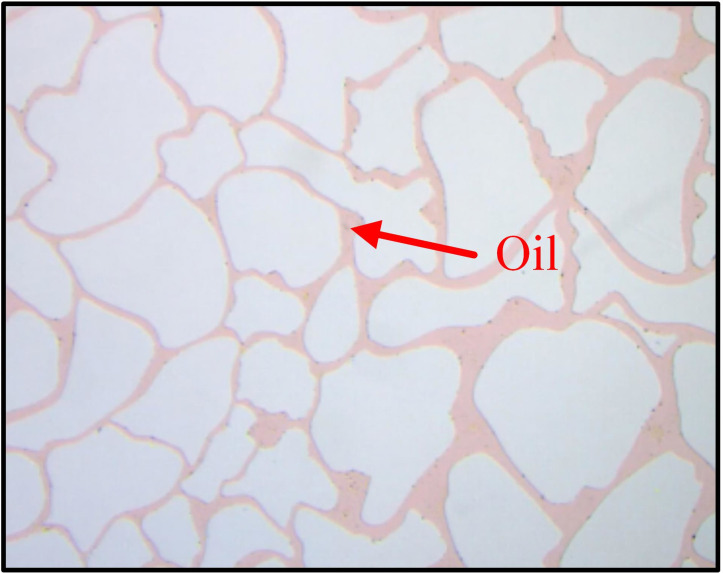
The glass-chip model saturated oil state.

(1) The glass-etched chip was placed in the microscopic visualization model gripper, vacuum-treated, and heated to 80°C.
(2) The initial enclosure pressure was set to 8 MPa, after which the chip was saturated with (Fig 9).
(3) CO_2_ was injected into the chip at a constant flow rate until no oil was produced at the outlet.
(4) The multiphase flow process was recorded using a visual observation system. The model was cleaned, and steps 1 to 3 were repeated for oil repelling experiments using different parameters.
(5) Adobe Photoshop was used to process the images captured during the experiments.

Table 3 shows the experimental protocol for the high-temperature, high-pressure microscopic visualization.

**Table 3:** The experimental program.

Case	Displacement process	Displacement pressure difference (MPa)	Injection rate (ul/min)	Confining pressure (MPa)
**1**	Immiscible CO_2_ flooding	1	0.5	8
**2**	Immiscible CO_2_ flooding	1	1	8
**3**	Immiscible CO_2_ flooding	1	2	8
**4**	Immiscible CO_2_ flooding	0.5	2	8
**5**	Immiscible CO_2_ flooding	1	2	13
**6**	Water flooding	1	2	13

## Results and discussion

Microscopic visualization experiments were conducted to analyze the flow characteristics and residual oil distribution of pore-scale multiphase flow in immiscible conditions, varying displacement pressure differences, injection velocities, and confining pressures, consequently elucidating the flow mechanism underlying CO_2_ displacement in complex pore structures. The four primary objectives of the high-temperature, high-pressure microscopic visualization experiments included the following: (1) The experimental microscopic visualization system was used to obtain the qualitative data of the pore-scale multiphase flow (oil and CO_2_) characteristics and CO_2_ recovery efficiency. (2) The ODE of CO_2_ injection and microscopic CO_2_-oil mechanism were investigated. (3) Image analysis technology was used to obtain the quantitative micro-data while the factors influencing EOR was evaluated. (4) The influence of the CO_2_ force action in the pores on the flow characteristics and the EOR mechanism was analyzed.

### 3.1. Immiscible CO _2_ injection experiments

**Fig 10:**
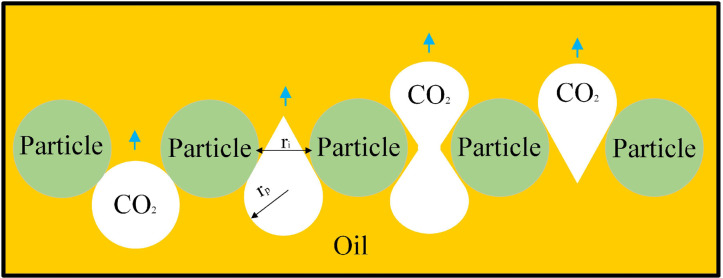
CO_2_ forces in pores capillary forces.

**Fig 11:**
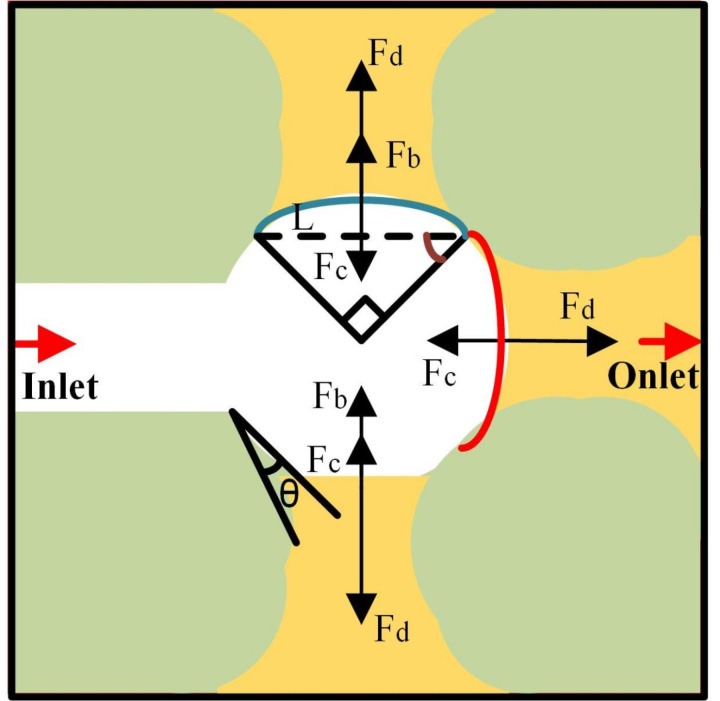
The force analysis of the CO_2_ contact surface.

When CO_2_ entered the crude oil-filled glass-etched chip, both the capillary force in the pore and the injection hydrodynamic force are displacement forces. However, when CO_2_ flooding occurs, oil is displaced by CO_2_ in the bellow and moves forward, so capillary force must be overcome. Therefore, capillary force is usually the resistance to CO_2_ displacement (Fig 10). The upper and lower CO_2_-oil contact surfaces were exposed to driving and capillary forces and buoyancy. The front contact surface was subjected to driving and capillary forces, while the CO_2_ was affected by the force within the pore (Fig 11).

The driving force was generated by the pressure difference between the CO_2_ and crude oil, which was the same as that between the inlet and outlet of the glass-etched chip [34]. The driving force was calculated using Equation 1 as follows:


$$F_{d}=(P^{n}-P^{w})\times \frac{R_{CO_{2}bubble}}{\cos(\frac{\pi }{4})}\times T_{thickness}$$ (1)

where $F_{d}$denotes the driving force (N), $P^{n}$signifies the non-wetting phase pressure (non-wetting phase is CO_2_) (MPa), $P^{w}$represents the wetting phase pressure (wetting phase is oil) (MPa), $R_{CO_{2}bubble}$denotes the CO_2_ radius (mm), and $T_{thickness}$is the CO_2_ thickness (mm).

The pressure discontinuity at the CO_2_-oil interface caused by surface tension produced a pressure difference in accordance with Young’s Laplace’s law:


$$P^{n}-P^{w}=S\sigma_{CO_{2}-oil}$$ (2)

where S- is the interface curvature. Due to the circular entry of CO_2_ into the pore, the interface curvature is$\frac{\text{1}}{R_{CO_{2}bubble}}$; $\sigma_{CO_{2}-oil}$is defined by the interfacial tension (N/mm).

The final driving force formula was obtained using Equations 1 and 2:


$$F_{d}=\sigma_{CO_{2}-oil}\times \cos(\frac{\pi }{4})\times T_{thickness}$$ (3)

CO_2_ was subjected to capillary pressure in the pores to generate capillary force, which acted on the CO_2_ contact surface, and was calculated as:


$$F_{c}=\frac{2\sigma_{CO_{2}-oil}(T_{thickness}+L)\cos(\theta )}{L\times T_{thickness}}\times \frac{R_{CO_{2}bubble}}{\cos(\frac{\pi }{4})}\times T_{thickness}$$ (4)

where $F_{c}$denotes the capillary force (N), *L*represents the arc length of the contact surface between the CO_2_ and the oil (mm), and *θ*signifies the contact angle of the CO_2_-oil in the glass-etched chip (°).

In addition, due to the difference in density between CO_2_ and crude oil, CO_2_ will be subjected to buoyancy force, and its own gravity is less than the buoyancy force$F_{b}$, so that the CO_2_-oil interface moves upward, and the direction of buoyancy is always upward. The size of its buoyancy force is calculated as follows:


$$F_{b}=(\rho_{oil}-\rho_{CO_{2}})\times g\times \pi R_{CO_{2}bubble}^{2}\times T_{thickness}$$ (5)

**Fig 12:**
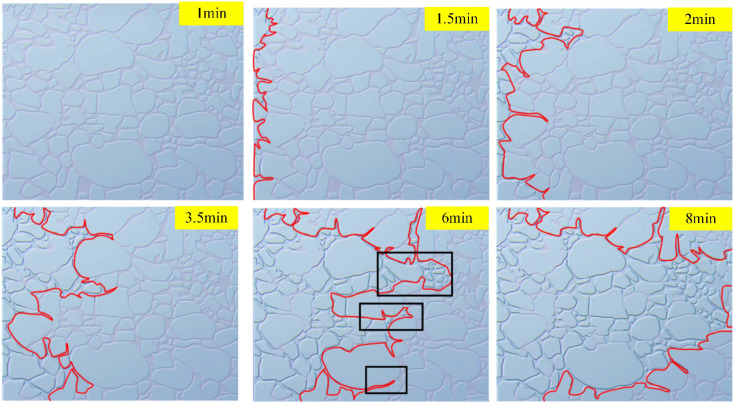
The fluid distribution of immiscible CO_2_ flooding at 8 MPa, 80°C, and different experimental times.

The microscopic visualization CO_2_ flooding experiment was conducted at a pressure of 8 MPa, a temperature of 80°C, and an injection speed of 2 ul/min. As shown in Fig 12, the resultant force acting on the upper contact surface of CO_2_-Oil is greater than that on the lower contact surface. So, the CO_2_ displayed a more rapid upward move than in other directions, with the formation of a dominant flow channel above the chip after 2 min. Due to the low viscosity of CO_2_, it flows relatively quickly and preferentially enters larger pore channels with smaller capillary forces, resulting in a fingering phenomenon. The subsequent force on the front CO_2_-oil contact surface exceeded that on the lower contact surface, resulting in the formation of beneficial flow channels in the central region of the chip after 3.5 min. During the continuous injection of CO_2_, the driving force continuously overcomes the capillary forces in smaller pore channels. At 6 min, the chip displayed distinct upper, middle, and lower flow channels. Finally, front merging occurred near the exit at 8 min, resulting in a collective breach of the right-side exit, with a recovery rate of 81.5%.

#### 3.1.1. Different injection rates.

**Fig 13:**
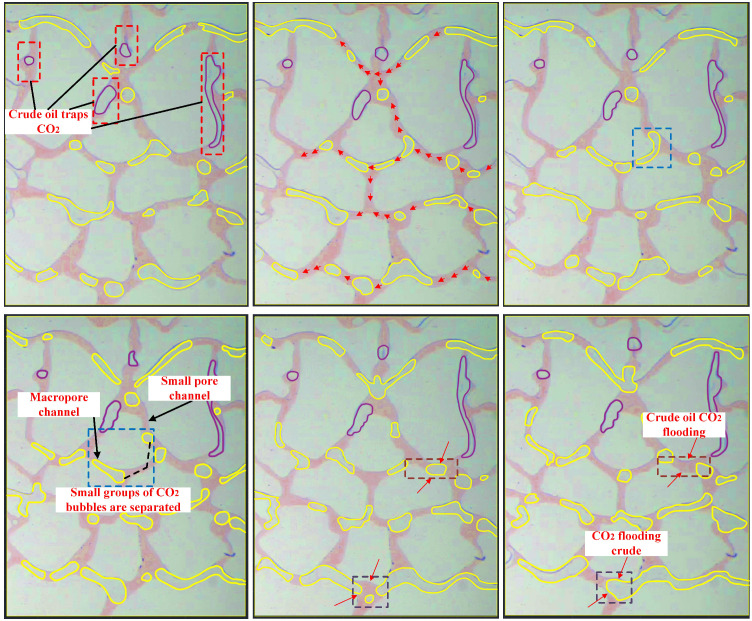
The displacement at an injection rate of 0.5 ul/min.

The temperature and pressure were 80°C and 8 MPa, and CO_2_ was injected at the injection speed of 0.5 ul/min. The oil displacement process is shown in Fig 13 (due to lighting problems, the chip presented different colors, but the experimental results were not affected). CO_2_ progressed in clusters of small bubbles and began to advance towards the macropore channels in the upper, middle, and lower directions as the displacement time increased. The small CO_2_ bubble clusters were separated when encountering a bifurcated channel. Most of them migrated forward into the macroporous channels. The capillary force in the narrow pore channel presented resistance, requiring the CO_2_ displacement force to exceed the capillary force to facilitate movement. Smaller CO_2_ bubbles were trapped in the narrow pores by crude oil, resulting in distinct CO_2_ flow stratification. Due to insufficient driving, smaller CO_2_ bubbles can be trapped within narrow pores by the surrounding crude oil. A higher CO_2_ injection quantity caused gradual CO_2_ accumulation in the narrow pores, increasing the volume and facilitating steady forward movement. This resulted in multiple oil-CO_2_-oil and CO_2_-oil-CO_2_ displacement phenomena.

**Fig 14:**
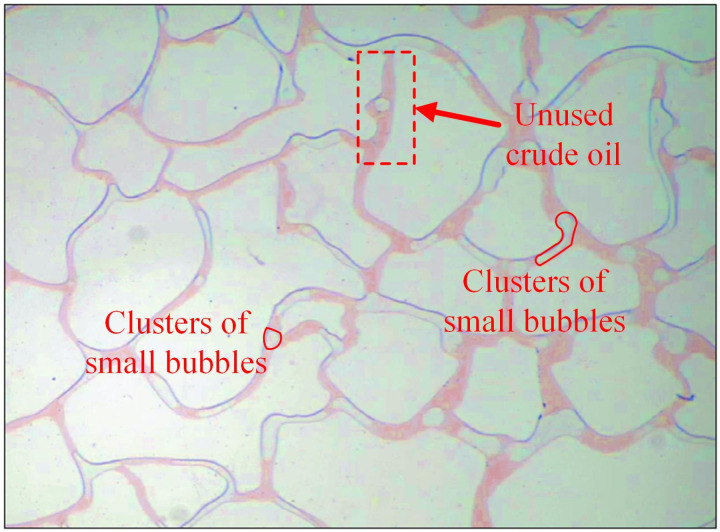
The displacement comparison diagram at injection rates of 0.5 ul/min.

**Fig 15:**
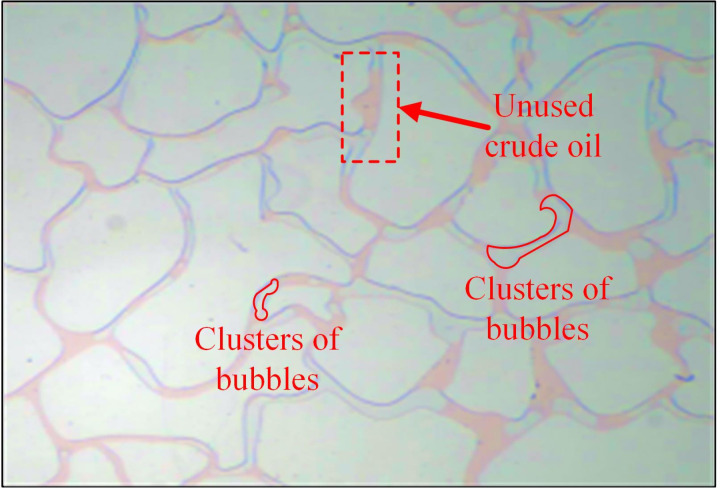
The displacement comparison diagram at injection rates of 1 ul/min.

**Fig 16:**
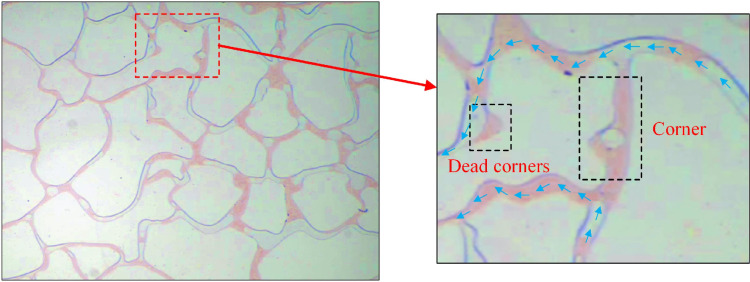
A crude oil injection rate of 1 ul/min.

At an injection speed of 0.5 ul/min, the residual oil at corners and dead corners in the pores was challenging to utilize and existed as island residual oil (Fig 14). Although the driving increased at an injection rate of 1 ul/min (Fig 15), but it is not enough to overcome the resistance in dead corners and turns. It was insufficient to overcome the capillary force at dead corners and corners, resulting in the crude oil in these areas being unusable (Fig 16).

**Fig 17:**
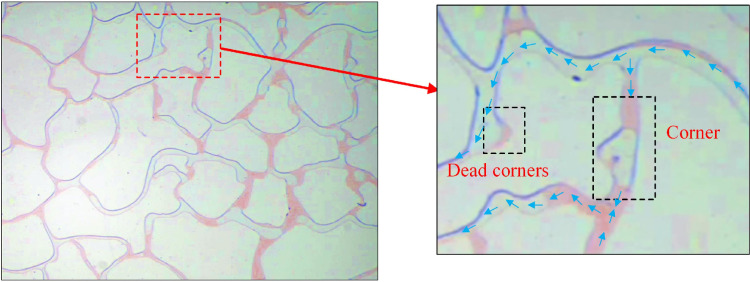
A crude oil injection rate of 2 ul/min.

**Fig 18:**
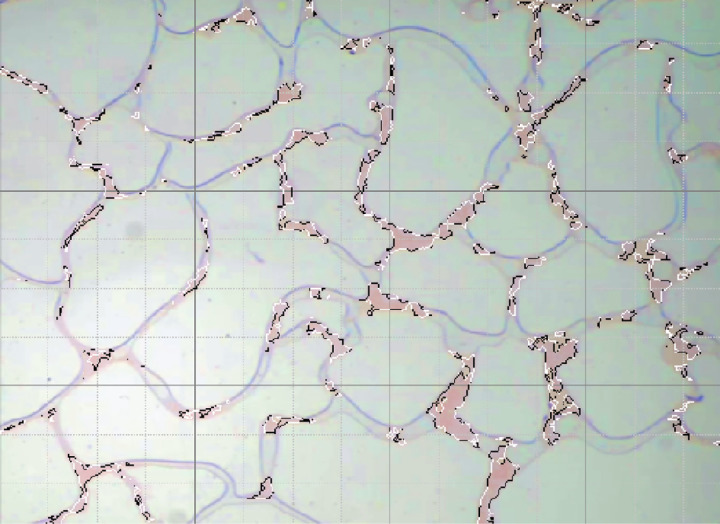
Extraction of the pigment points from the final oil image during CO_2_ flooding.

**Fig 19:**
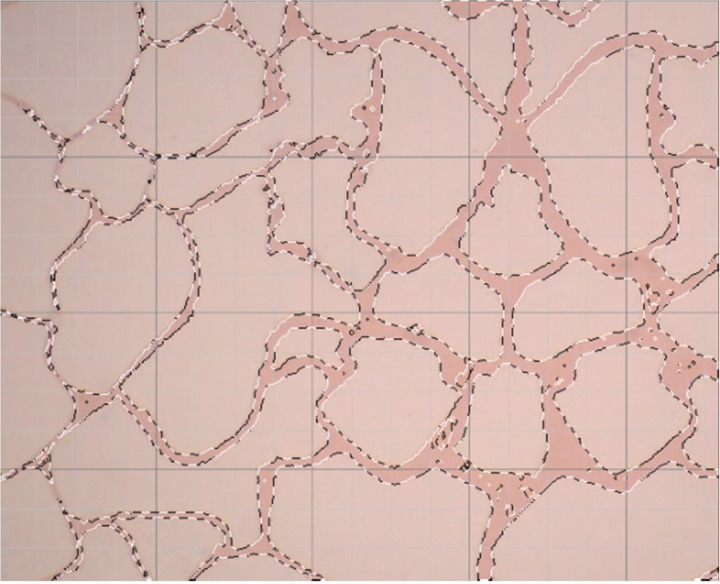
The pigment point of the oil extracted at saturation.

**Fig 20:**
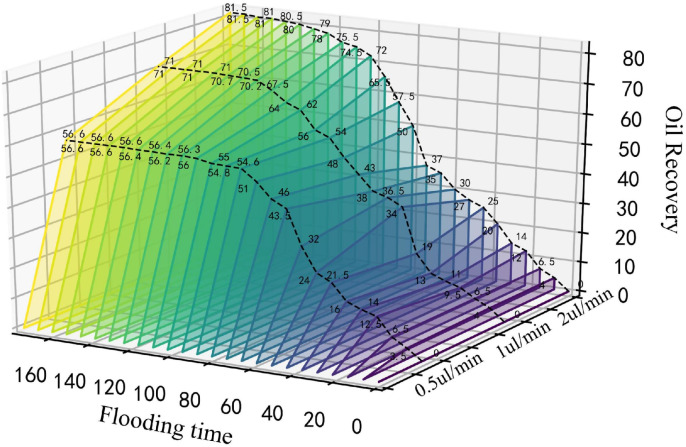
The recovery curves at different injection rates.

At an injection rate of 2 ul/min, the CO_2_ formed large bubbles and flowed continuously in the pores. The subsequent driving was sufficient to overcome the capillary force at dead corners and corners, causing partial, more effective residual oil displacement (Fig 17). This was because the capillary force generated more significant CO_2_ flow resistance when the CO_2_ entered the narrow pores during displacement. Therefore, a more substantial driving was necessary to promote unrestricted CO_2_ flow. This enabled the dispersion of the CO_2_ bubble clusters in different flow channels and into smaller pore channels, consequently increasing the sweep range and recovery rate. Adobe Photoshop was used to preprocess the final image, while the Image J software was used to analyze the gray values of the image. The residual oil was converted into red pixels, while the pigment points of the oil phase were extracted and calculated (Fig 18). Additionally, the oil pigment area was extracted in conjunction with saturated oil (Fig 19). The recovery efficiency was determined at different injection rates (Fig 20).

#### 3.1.2. Different displacement confining pressures.

**Fig 21:**
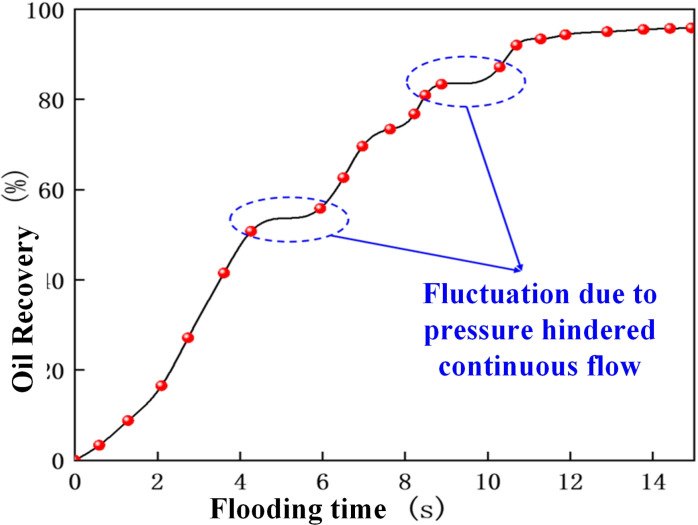
The CO_2_ flooding oil recovery curve at a confining pressure of 13 MPa.

**Fig 22:**
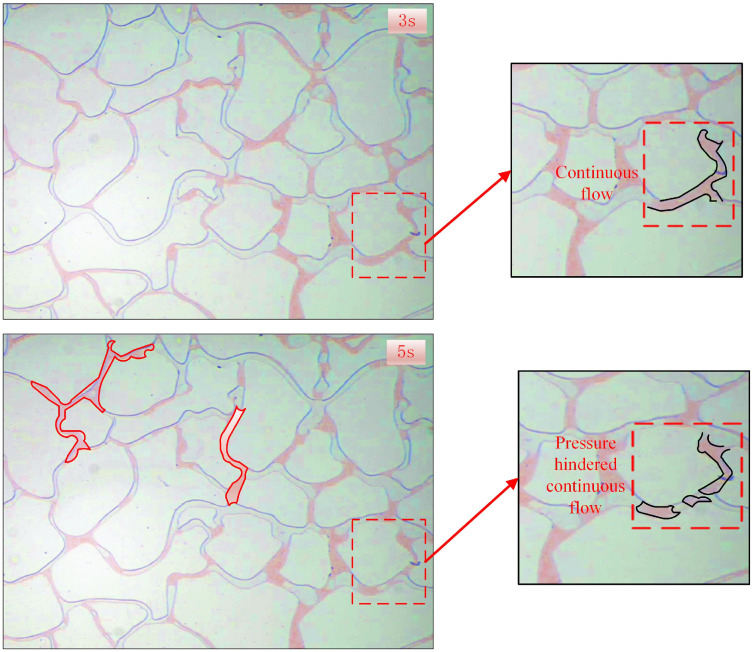
The spatial and temporal CO_2_ distribution at a confining pressure of 13 MPa.

The CO_2_ displacement experiment was conducted at an injection rate of 2 ul/min, a displacement pressure difference of 1 MPa, and a confining pressure of 13 MPa, while the recovery curves were recorded at different time (Fig 21). As shown in Fig 22, a higher confining pressure increased the CO_2_ pressure in the chip and significant amounts of banded residual oil adhered to both sides of the pore channel, restricting the CO_2_ flow in the narrow pores, while the small, separated bubble clusters passed through the small pore channels. The high confining pressure acting on the glass chip hindered continuous CO_2_ flow, causing unstable CO_2_ displacement front fluctuations. This is because under the action of large confining pressure, due to the extremely low interfacial tension of CO_2_, it passes through the crude oil in the pore channel, resulting in a flow effect similar to the gas channeling effect. Consequently, the recovery curves displayed displacement fluctuations (Fig 21).

Therefore, the confining pressure can be increased to improve the CO_2_ density, reduce the influence of buoyancy, expand the CO_2_ sweep area, and increase the recovery efficiency by 94.4%.

#### 3.1.3. Displacement pressure differences.

**Fig 23:**
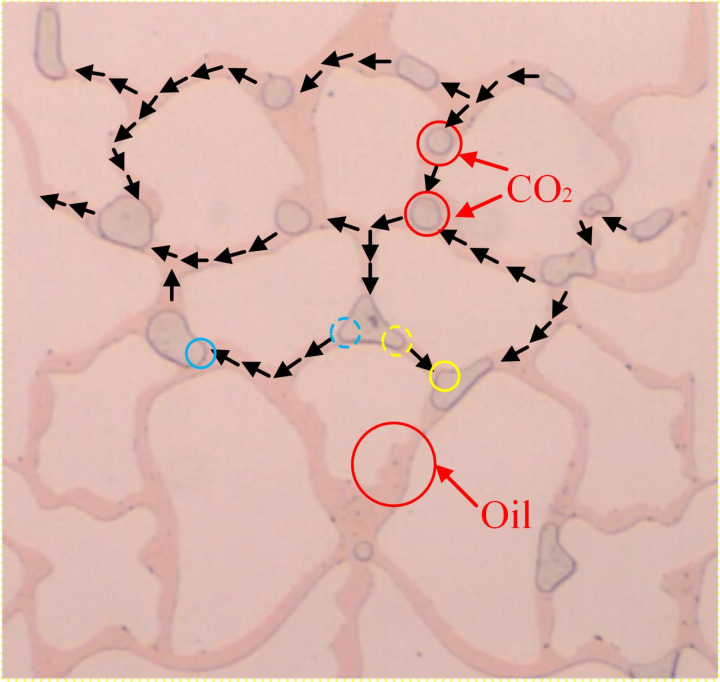
The CO_2_ flooding at 0.5MPa displacement pressure differences.

The temperature and pressure were 80°C, 8 MPa, the displacement pressure difference was 0.5 MPa, and the CO_2_ injection rate was 2 ul/min. As shown in Fig 23, the CO_2_ was mainly concentrated in the large pore channel during crude oil displacement, with only a small quantity entering the small pore channel, resulting in a limited displacement area. Minimal CO_2_ accumulation and a decline in the flow rate was evident. When the CO_2_ encountered small pores during the flow process, it separated into smaller volumes and continued to flow forward, subsequently amalgamating again in large pores to propel the oil forward. A lower displacement pressure difference reduced the CO_2_ flow displacement force. The capillary force impeded CO_2_ migration in the small pore channel, restricting its entry. Therefore, at a low displacement pressure difference, the oil displacement efficacy of the CO_2_ entering the large pore channel surpassed that of the small pore channel.

**Fig 24:**
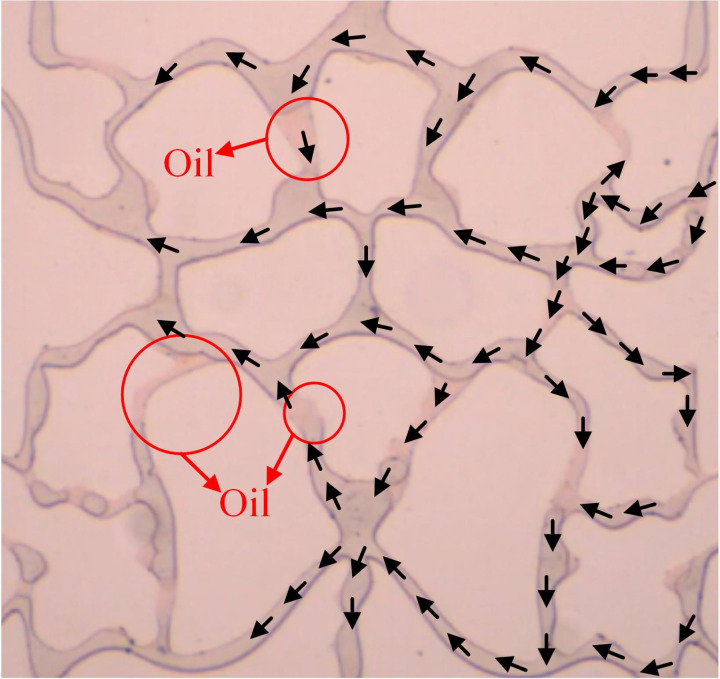
The CO_2_ flooding at 1MPa displacement pressure differences.

**Fig 25:**
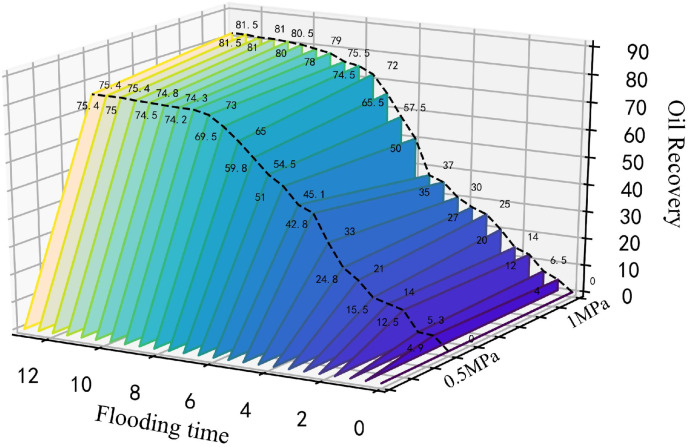
The recovery curves of various displacement pressure differences.

As shown in Fig 24, a displacement pressure difference of 1 MPa significantly increased the CO_2_ displacement area, accumulation volume, flow velocity, oil displacement efficacy, ability to overcome capillary forces, and the recovery efficiency by 6.1% (Fig 25).

### Water injection experiments

**Fig 26:**
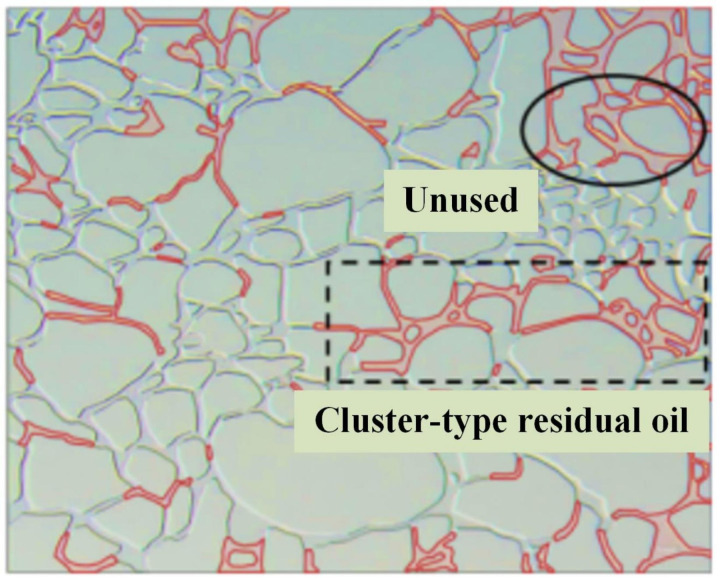
The water displacement fluid distribution at 13 MPa and an injection speed of 2 ul/min.

**Fig 27:**
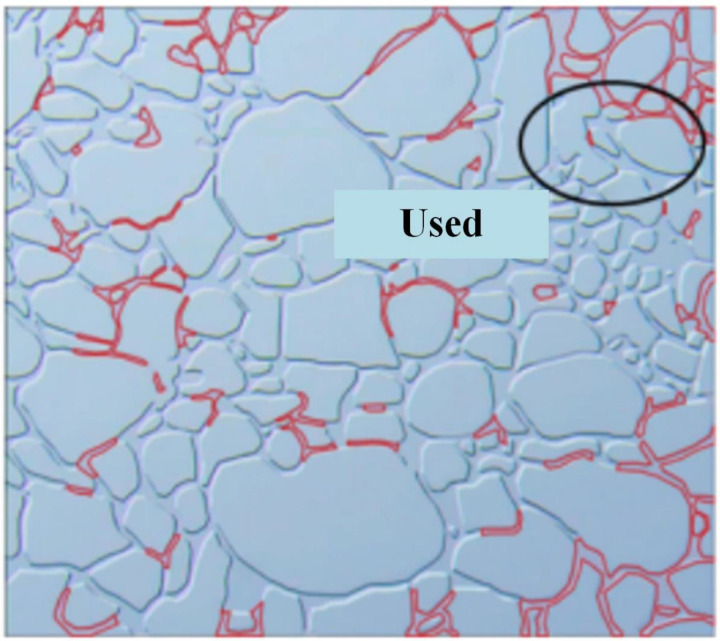
The CO_2_ displacement fluid distribution at 13 MPa and an injection speed of 2 ul/min.

**Fig 28:**
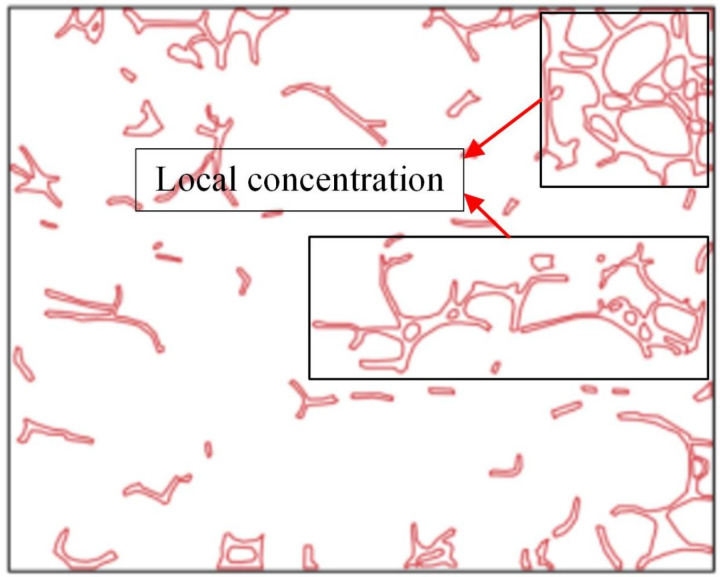
The quantitative gray value assessment of the images after water flooding, using the Image J software.

**Fig 29:**
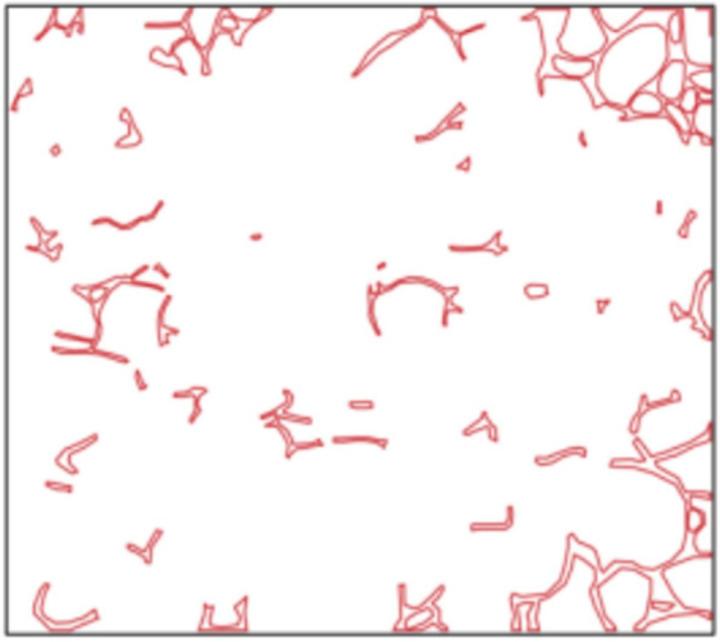
The quantitative gray value assessment of the images after CO_2_ flooding, using the Image J software.

**Fig 30:**
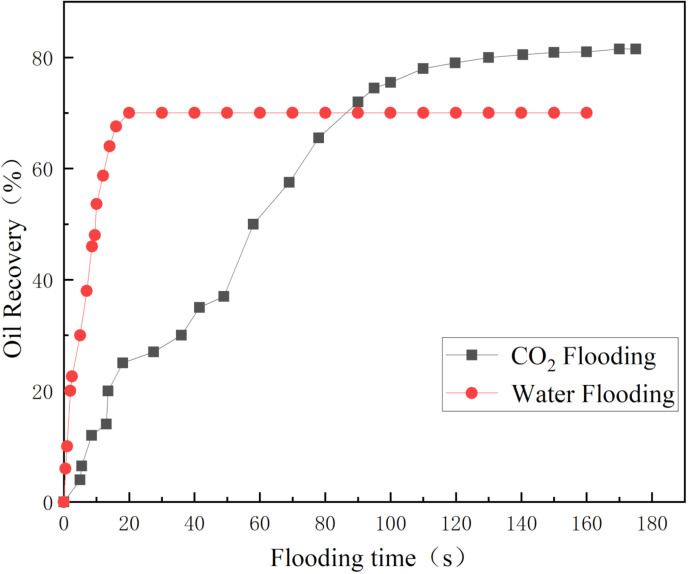
The oil recovery after water and CO_2_ flooding.

The microvisual water flooding and CO_2_ flooding microfluidic experiments were conducted at a pressure of 13 MPa, a temperature of 80°C, and an injection speed of 2 ul/min. Both capillary and injection hydrodynamic forces are forms of displacement forces. However, since the water viscosity was substantially higher than that of CO_2_, it was necessary to overcome the viscous force when the water entered the pore channel. This reduced the displacement force of the water relative to CO_2_, complicating crude oil movement in the pore throat (Fig 26). Therefore, CO_2_ was more suitable for oil displacement. Most of the residual oil captured after water flooding was present in the pore channel as clusters (Fig 27). Adobe Photoshop was used for final image preprocessing, while the Image J software was used to determine the gray values, as shown in Figs 28 and 29. The residual oil was represented by red pixels, while the remainder was denoted by white pixels. Additionally, the water and CO_2_ flooding oil recovery rates were calculated (Fig 30).

## Conclusion

This study improves the microscopic visualization flow system, enhancing the working temperature and pressure and increasing data recovery recording capacity. The multiphase flow characteristics, oil-CO_2_ transport, and CO_2_ displacement efficacy are analyzed via CO_2_ displacement experiments at different pore scales.

(1) The equipment used for microscopic flow system visualization is enhanced, while the optimized working temperature and pressure reach 120°C and 25 MPa, respectively. The glass-etched chip is heated and pressurized to experimental conditions using a high-pressure containment kettle and temperature control system.
(2) The CO_2_ is affected by buoyancy and capillary force during immiscible CO_2_ flooding, restricting access to the crude oil in small pores, corners, and dead corners. This results in a cut-off phenomenon, a reduced sweep area, and limited oil recovery.
(3) The immiscible CO_2_ flooding experiment reveals differences in the multiphase flow characteristics between CO_2_ and water flooding. The pore size, injection velocity, displacement pressure difference, and confining pressure affect the multiphase flow process. The complex flow characteristics of these multiphase flows affect residual oil distribution, formation, and sweep area, ultimately revealing the pore-scale CO_2_ flooding flow mechanism.
(4) Since the water viscosity far exceeds that of CO_2_ during water flooding, it is necessary to overcome the viscous force when water enters the pore channel. This reduces displacement force of the water relative to CO_2_, complicating crude oil movement in the pore throat. Consequently, the recovery rate is 11.5% lower than that of CO_2_ flooding. A higher CO_2_ injection speed increases the displacement force, enhancing the capacity to overcome the capillary force. This allows the CO_2_ to enter smaller pore channels and increases the sweep range, while enhancing the recovery rate by 24.9%. Adequately increasing the displacement pressure difference improves the CO_2_ flow capacity and oil displacement efficacy, while enhancing the recovery rate by 6.1%. A higher confining pressure causes a significant quantity of banded residual oil to adhere to both sides of the pore channel, resulting in the cut-off flow phenomenon. Therefore, appropriately increasing the confining pressure improves the CO_2_ density, reduces the influence of buoyancy, expands the CO_2_ sweep area, and increases the recovery rate by 12.9%.
(5) This study examines the pore-scale flow mechanism of immiscible CO_2_ flooding and recommends the reasonable design of parameters for CO_2_ immiscible injection in Chinese reservoirs to improve oil recovery. It also offers theoretical guidelines for future research on the integration of CO_2_ storage and utilization.

## References

[1] Pelto MS. Impact of climate change on North Cascade Alpine Glaciers, and Alpine Runoff. Northwest Sci. 2008;82(1):65–75. doi: 10.3955/0029-344x-82.1.65

[2] Diffenbaugh NS, Burke M. Global warming has increased global economic inequality. Proc Natl Acad Sci U S A. 2019;116(20):9808–13. doi: 10.1073/pnas.1816020116 31010922 PMC6525504

[3] Rehman A, Ma H, Irfan M, Ahmad M. Does carbon dioxide, methane, nitrous oxide, and GHG emissions influence the agriculture? Evidence from China. Environ Sci Pollut Res Int. 2020;27(23):28768–79. doi: 10.1007/s11356-020-08912-z 32347504

[4] Liu B. The basis, challenges and policy path for China to achieve carbon peak and carbon neutral goals. Monthly price. 2021;534.

[5] Yu H, Lu X, Fu W, Wang Y, Xu H, Xie Q, et al. Determination of minimum near miscible pressure region during CO2 and associated gas injection for tight oil reservoir in Ordos Basin, China. Fuel. 2020;263:116737. doi: 10.1016/j.fuel.2019.116737

[6] Zendehboudi S, Khan A, Carlisle S, Leonenko Y. Ex situ dissolution of CO2: a new engineering methodology based on mass-transfer perspective for enhancement of CO2 sequestration. Energy Fuels. 2011;25(7):3323–33. doi: 10.1021/ef200199r

[7] Alhosani A, Lin Q, Scanziani A, Andrews E, Zhang K, Bijeljic B, et al. Pore-scale characterization of carbon dioxide storage at immiscible and near-miscible conditions in altered-wettability reservoir rocks. Int J Greenhouse Gas Control. 2021;105:103232. doi: 10.1016/j.ijggc.2020.103232

[8] Zhao X, Liao X, Wang W, Chen C, Rui Z, Wang H. The CO2 storage capacity evaluation: methodology and determination of key factors. J Energy Inst. 2014;87(4):297–305. doi: 10.1016/j.joei.2014.03.032

[9] Bui M, Adjiman CS, Bardow A, Anthony EJ, Brown S, et al. Carbon capture and storage (CCS): the way forward. Energy Environ. 2018;11(5):1062–176. doi: 10.1039/C7EE02342A

[10] Xing X, Bian X-Q, Zhang J, Zeng Y, Li J. A data-driven dual-optimization hybrid machine learning model for predicting carbon dioxide trapping efficiency in saline aquifers: Application in carbon capture and storage. Geoenergy Sci Eng. 2024;243:213363. doi: 10.1016/j.geoen.2024.213363

[11] Zuloaga P, Yu W, Miao J, Sepehrnoori K. Performance evaluation of CO2 Huff-n-Puff and continuous CO_2_ injection in tight oil reservoirs. Energy. 2017;134:181–92. doi: 10.1016/j.energy.2017.06.028

[12] Hassanpouryouzband A, Yang J, Tohidi B, Chuvilin E, Istomin V, Bukhanov B. Geological CO2 capture and storage with flue gas hydrate formation in frozen and unfrozen sediments: method development, real time-scale kinetic characteristics, efficiency, and clathrate structural transition. ACS Sustainable Chem Eng. 2019;7(5):5338–45. doi: 10.1021/acssuschemeng.8b06374

[13] Zhao Y, Zhang Y, Lei X, Zhang Y, Song Y. CO2 flooding enhanced oil recovery evaluated using magnetic resonance imaging technique. Energy. 2020;203:117878. doi: 10.1016/j.energy.2020.117878

[14] Hassanpouryouzband A, Joonaki E, Edlmann K, Haszeldine RS. Offshore geological storage of hydrogen: is this our best option to achieve net-zero? ACS Energy Lett. 2021;6(6):2181–6. doi: 10.1021/acsenergylett.1c00845

[15] Zhang L, Tan X, Tian X, Jiao Y, Zhang W, Shu X, et al. Inspirations from field-reservoir co2 flooding with different miscible degrees under cross-scale oil reservoir conditions. ACS Omega. 2024;9(13):14692–703. doi: 10.1021/acsomega.3c08433 38585085 PMC10993391

[16] Shyeh-Yung J-G. J. Mechanisms of miscible oil recovery: effects of pressure on miscible and near-miscible displacements of oil by carbon dioxide. SPE Annual Technical Conference and Exhibition. Dallas, Texas: Society of Petroleum Engineers; 1991. No. 22651.

[17] Li L, Su Y, Sheng JJ, Hao Y, Wang W, Lv Y, et al. Experimental and numerical study on CO2 sweep volume during CO2 Huff-n-Puff enhanced oil recovery process in shale oil reservoirs. Energy Fuels. 2019;33(5):4017–32. doi: 10.1021/acs.energyfuels.9b00164

[18] Hamza A, Hussein IA, Al-Marri MJ, Mahmoud M, Shawabkeh R, Aparicio S. CO2 enhanced gas recovery and sequestration in depleted gas reservoirs: a review. J Petrol Sci Eng. 2021;196:107685. doi: 10.1016/j.petrol.2020.107685

[19] Lv G, Li Q, Wang S, Li X. Key techniques of reservoir engineering and injection–production process for CO2 flooding in China’s SINOPEC Shengli Oilfield. J CO_2_ Util. 2015;11:31–40. doi: 10.1016/j.jcou.2014.12.007

[20] Li Z, Su Y, Li L, Hao Y, Wang W, Meng Y, et al. Evaluation of CO2 storage of water alternating gas flooding using experimental and numerical simulation methods. Fuel. 2022;311:122489. doi: 10.1016/j.fuel.2021.122489

[21] Jia B, Tsau J-S, Barati R. A review of the current progress of CO2 injection EOR and carbon storage in shale oil reservoirs. Fuel. 2019;236:404–27. doi: 10.1016/j.fuel.2018.08.103

[22] Andrew M, Bijeljic B, Blunt MJ. Pore‐by‐pore capillary pressure measurements using X‐ray microtomography at reservoir conditions: Curvature, snap‐off, and remobilization of residual CO2. Water Resourc Res. 2014;50(11):8760–74. doi: 10.1002/2014wr015970

[23] Alhosani A, Scanziani A, Lin Q, Pan Z, Bijeljic B, Blunt MJ. In situ pore-scale analysis of oil recovery during three-phase near-miscible CO2 injection in a water-wet carbonate rock. Adv Water Resourc. 2019;134:103432. doi: 10.1016/j.advwatres.2019.103432

[24] Scanziani A, Singh K, Bultreys T, Bijeljic B, Blunt MJ. In situ characterization of immiscible three-phase flow at the pore scale for a water-wet carbonate rock. Adv Water Resourc. 2018;121:446–55. doi: 10.1016/j.advwatres.2018.09.010

[25] Silin D, Patzek TW, Benson SM. A one-dimensional model of vertical gas plume migration through a heterogeneous porous medium. Int J Greenhouse Gas Control. 2009;3(3):300–10. doi: 10.1016/j.ijggc.2008.09.003

[26] Perrin J-C, Benson S. An Experimental study on the influence of sub-core scale heterogeneities on CO2 distribution in reservoir rocks. Transp Porous Med. 2009;82(1):93–109. doi: 10.1007/s11242-009-9426-x

[27] Krevor SCM, Pini R, Li B, Benson SM. Capillary heterogeneity trapping of CO2 in a sandstone rock at reservoir conditions. Geophys Res Lett. 2011;38(15). doi: 10.1029/2011gl048239

[28] Nguyen P, Carey JW, Viswanathan HS, Porter M. Effectiveness of supercritical-CO2 and N2 huff-and-puff methods of enhanced oil recovery in shale fracture networks using microfluidic experiments. Appl Energy. 2018;230:160–74. doi: 10.1016/j.apenergy.2018.08.098

[29] Tang Y, Hou C, He Y, Wang Y, Chen Y, Rui Z. Review on pore structure characterization and microscopic flow mechanism of CO2 flooding in Porous Media. Energy Tech. 2020;9(1). doi: 10.1002/ente.202000787

[30] Tang Y, Su Z, He J, Yang F. Numerical simulation and optimization of enhanced oil recovery by the in situ generated CO2Huff-n-Puff process with compound surfactant. J Chem. 2016;2016:1–13. doi: 10.1155/2016/6731848

[31] Jiang J, Rui Z, Hazlett R, Lu J. An integrated technical-economic model for evaluating CO2 enhanced oil recovery development. Appl Energy. 2019;247:190–211. doi: 10.1016/j.apenergy.2019.04.025

[32] Sontheimer-Phelps A, Hassell BA, Ingber DE. Modelling cancer in microfluidic human organs-on-chips. Nat Rev Cancer. 2019;19(2):65–81. doi: 10.1038/s41568-018-0104-6 30647431

[33] Talebkeikhah M, Nait Amar M, Naseri A, Humand M, Hemmati-Sarapardeh A, Dabir B, et al. Experimental measurement and compositional modeling of crude oil viscosity at reservoir conditions. J Taiwan Inst Chem Eng. 2020;109:35–50. doi: 10.1016/j.jtice.2020.03.001

[34] Hao Y, Li Z, Su Y, Kong C, Chen H, Meng Y. Experimental investigation of CO2 storage and oil production of different CO2 injection methods at pore-scale and core-scale. Energy. 2022;254:124349. doi: 10.1016/j.energy.2022.124349

